# Quantitative histopathologic profiling of arterial dissection-related thrombi in acute ischemic stroke: etiological comparisons

**DOI:** 10.3389/fneur.2025.1640562

**Published:** 2025-08-20

**Authors:** Gang Cai, XuMing Fang, Jun Li, YuanHua Cheng, YanHui Zhang, Lan Chu

**Affiliations:** ^1^Department of Neurology, Affiliated Hospital of Guizhou Medical University, Guiyang, China; ^2^Institute of Neuroscience, Soochow University, Suzhou, China; ^3^Department of Pathology, Affiliated Hospital of Guizhou Medical University, Guiyang, China; ^4^Department of Neurology, Beijing Amcare Hospital, Beijing, China

**Keywords:** acute ischemic stroke, thrombectomy, arterial dissection, thrombus, etiology

## Abstract

**Introduction:**

Arterial dissection is an important etiology of stroke in young adults and may demonstrate distinct thrombus characteristics. While most studies have focused on compositional differences between cardioembolic (CE) and non-cardioembolic thrombi, systematic analyses of dissection-related thrombi remain scarce. This study characterized the compositions of dissection thrombi, compared them with those of non-dissection thrombi, and explored compositional variations among stroke etiologies.

**Materials and methods:**

We retrospectively analyzed the clinical data and thrombi of 230 patients with acute ischemic stroke (AIS). The thrombi were stained with hematoxylin and eosin (H&E) and digitally scanned, and their composition was analyzed based on quantified red blood cell (RBC), fibrin/other component (F/O), and white blood cell (WBC) ratios and the total thrombus area. Comparisons were made between dissection-related AIS and non-dissection etiologies (large artery atherosclerosis [LAA], CE, stroke of other determined etiology [SOE], and stroke of undetermined etiology [SUE]).

**Results:**

Among the 230 patients with acute ischemic stroke, 16 (7.0%) had dissection-related stroke and 214 (93.0%) had non-dissection stroke (59 LAA, 124 CE, 2 SOE, and 29 SUE). Compared to those in the non-dissection group, the patients in the dissection group were significantly younger and had a lower prevalence of hypertension and atrial fibrillation but higher tandem lesion frequency (all *p* < 0.05). Regarding the thrombus characteristics, no significant differences were observed in the RBC, F/O, or WBC ratios between the dissection and non-dissection groups or subgroups. However, the LAA thrombi were significantly smaller than those in the dissection, CE, and SUE groups (*p* < 0.05).

**Conclusion:**

The H&E-based thrombus composition analysis did not differentiate arterial dissection from other stroke etiologies. LAA thrombi exhibited the smallest thrombus burden among the major stroke subtypes.

## 1 Introduction

The widespread adoption of mechanical thrombectomy has enabled the analysis of thrombi retrieved from patients with acute ischemic stroke (AIS). There is growing interest in the relationship between thrombi composition, including red blood cell (RBC), fibrin/other components (F/O), white blood cell (WBC) ratios, and stroke etiology (1). Identifying the etiology of stroke is crucial for secondary prevention. Arterial dissection, a prevalent cause of stroke in young patients (2), may exhibit diagnostically significant thrombi characteristics. Studies have primarily focused on the compositional differences among cardioembolism (CE), large artery atherosclerosis (LAA), and stroke of undetermined etiology (SUE), revealing significant heterogeneity (3, 4). However, systematic investigations that specifically address thrombi compositions in cases of arterial dissection are scarce. Therefore, this study aimed to characterize dissection thrombi compositions and compare them with those of non-dissection thrombi to explore the differences in thrombi compositions across various stroke etiologies.

## 2 Materials and methods

### 2.1 Patients

**Figure 1:**
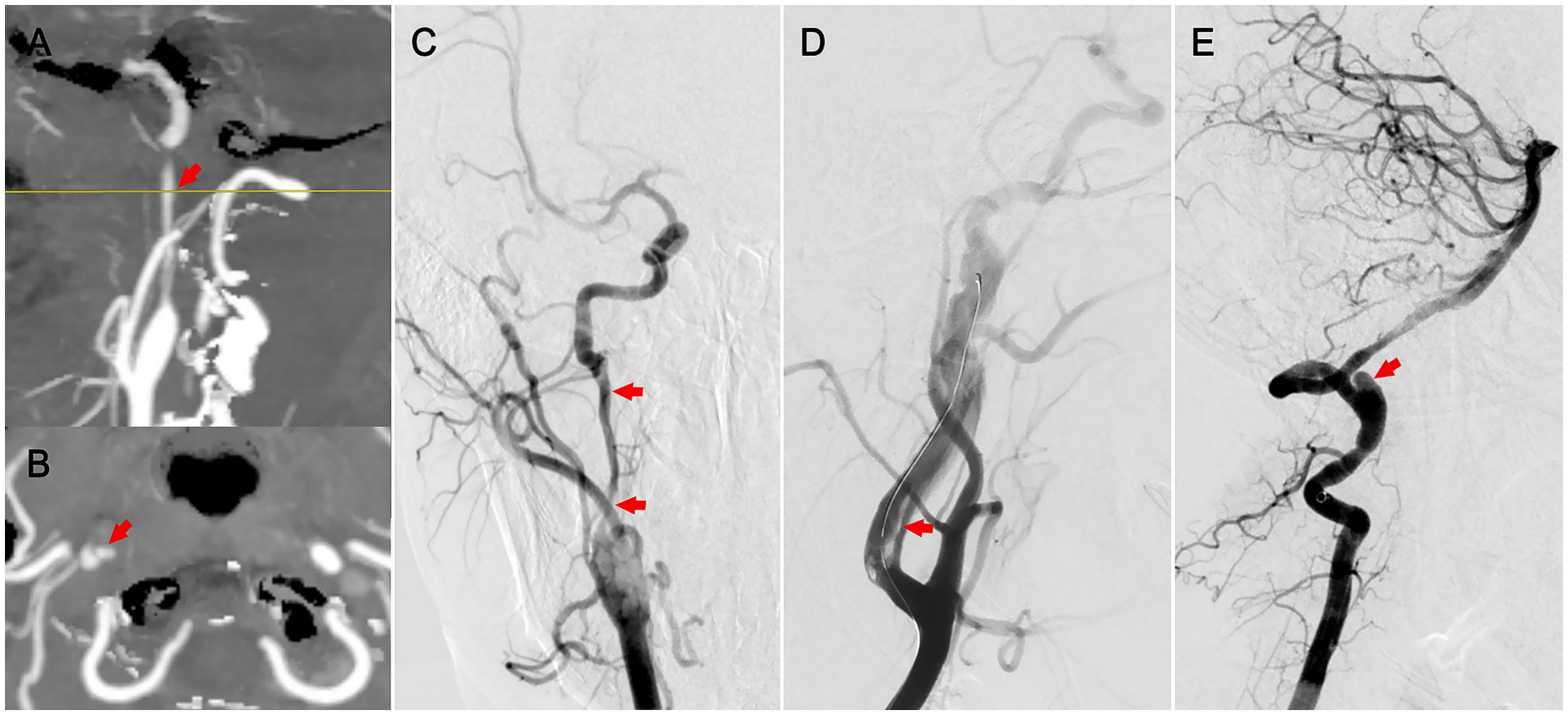
Radiographic features of cervical artery dissection. **(A)** Sagittal CTA indicating long-segment stenosis of the internal carotid artery (patient 1). **(B)** Axial CTA showing a double lumen (patient 1). **(C)** Anteroposterior DSA demonstrating long-segment stenosis with dissection (patient 1). **(D)** Lateral DSA revealing an intimomedial flap in the carotid artery (patient 2). **(E)** Lateral DSA showing a dissecting aneurysm in the vertebral artery (patient 3).

We reviewed the records of 331 patients with AIS who underwent emergency endovascular treatment at the Affiliated Hospital of Guizhou Medical University between June 2021 and June 2024. After excluding 88 patients, including 76 LAA, 5 CE, 4 carotid dissections, 1 stroke of other determined etiologies (SOE), and 2 SUE, and 13 patients for whom thrombus specimens were unavailable, 230 patients (69.5%) were included in the final analysis (Supplementary Figure 1). Clinical data were extracted from a prospectively maintained registry database and the hospital’s electronic medical record system. Stroke etiology was classified using the Trial of ORG 10172 in Acute Stroke Treatment (TOAST) criteria (5). Patients with arterial dissection-related stroke were assigned to the dissection group, whereas those with non-dissection etiologies, including LAA, CE, SOE, and SUE, constituted the non-dissection group. The dissection of the cervical carotid or vertebral artery requires radiological intervention via CTA or DSA, demonstrating the presence of a mural hematoma, dissecting aneurysm, long tapering stenosis, intimal flap, double lumen, or occlusion >2 cm above the carotid bifurcation and revealing a dissecting aneurysm or long tapering stenosis after recanalization (6, 7) (Figure 1). Patients who underwent iatrogenic artery dissection secondary to endovascular procedures were excluded.

### 2.2 Thrombus processing and analysis

**Figure 2:**
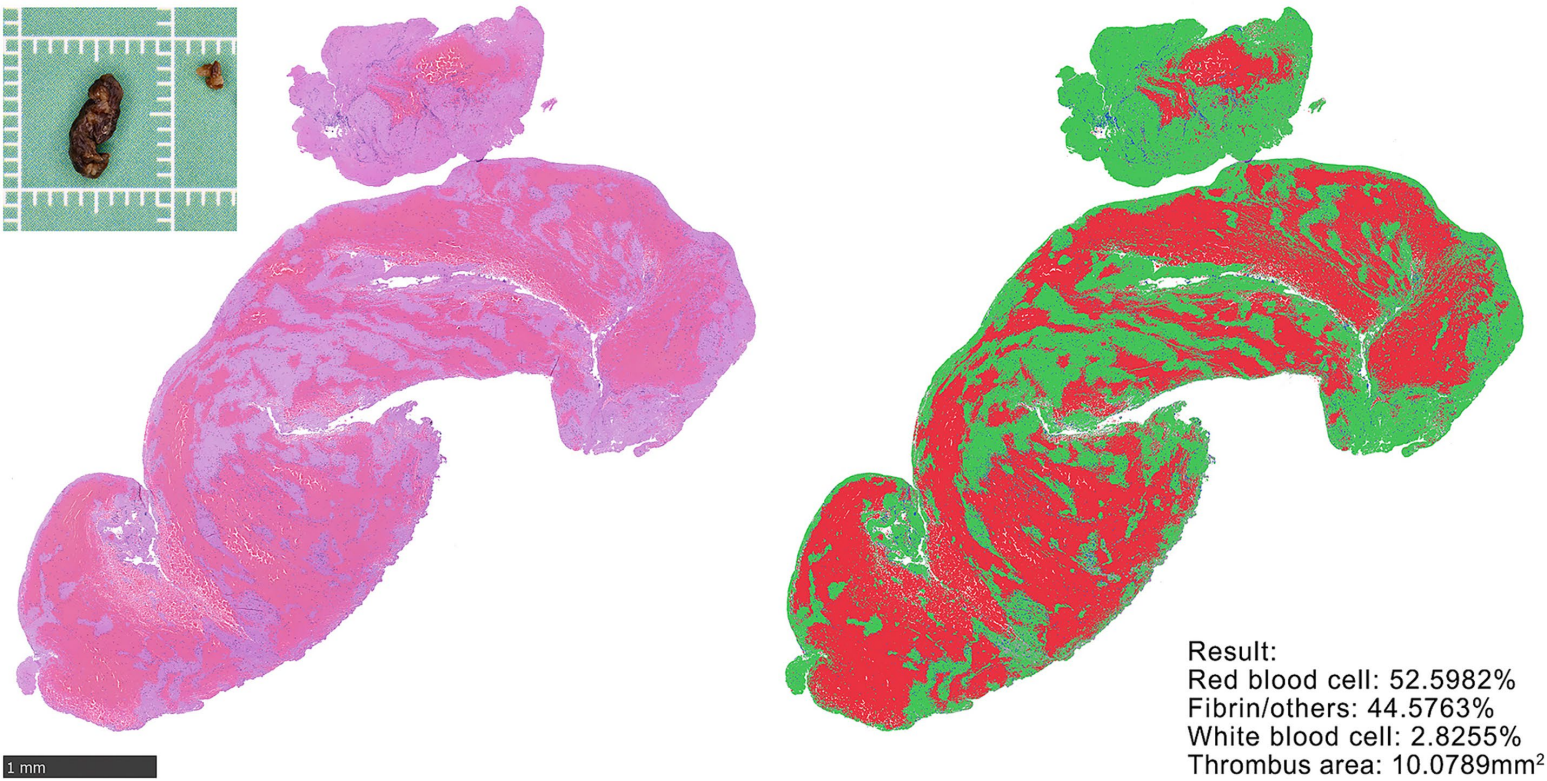
H&E staining and quantification of thrombus. Gross specimen of retrieved thrombus (upper left). Representative HE-stained section (left). Thrombus composition quantified using the Orbit Image Analysis model. Calculation results (lower right). Thrombus area was quantified using an area quantification model.

The thrombi were collected and preserved according to the standardized protocol described by Staessens et al. (8). Each thrombus retrieved per procedural pass was fixed in 10% phosphate-buffered formalin solution for 24 h, dehydrated, and embedded in paraffin. From each paraffin block, representative sections (2–5 μm) were cut and stained with hematoxylin and eosin (H&E). All the sections were digitized using a Hamamatsu (C13210) whole-slide scanner. A researcher with over 10 years of expertise in histopathological analysis independently quantified the thrombus components using Orbit Image Analysis software (version 3.64) (9). For each case, three validated computational models were applied, and the mean values were calculated for the RBC ratio, F/O ratio, WBC ratio, and total histopathological section area (Figure 2). Thrombi retrieved from the same patient during sequential passes were combined and analyzed as a single composite sample.

### 2.3 Statistical analysis

Categorical data are presented as frequencies (percentages), and continuous variables are expressed as medians (interquartile range). Group comparisons for categorical variables were conducted using χ^2^ or Fisher’s exact tests, with *post hoc* Bonferroni correction for pairwise comparisons when the overall test was significant. For continuous variables, Mann–Whitney *U*-tests were used for two-group comparisons, and Kruskal–Wallis tests with Dunn’s correction were employed for multi-group analyses. The SOE subgroup (*n* = 2) was excluded from inferential statistics due to methodological constraints but was included in descriptive reporting. All statistical analyses were performed using IBM SPSS Statistics (v20.0), with two-tailed *p* < 0.05 indicating significance.

## 3 Results

Among the 230 patients with AIS, 16 (7.0%) had arterial dissection-related stroke, and 214 (93.0%) had non-dissection etiologies. The non-dissection group comprised 59 LAA cases, 124 CE cases, two SOE cases (patients with confirmed non-dissection etiologies: 1 carotid stent thrombosis and 1 systemic lupus erythematosus-related stroke), and 29 SUE cases. The patients in the dissection group were significantly younger than those in the non-dissection group (45 vs. 66 years, *p* < 0.001) and exhibited a lower prevalence of hypertension (12.5% vs. 53.5%, *p* = 0.002) and atrial fibrillation (0% vs. 50.5%, *p* < 0.001). Tandem lesions were predominantly observed in the dissection group (100% vs. 18.2%, *p* < 0.001). The proportion of extracranial artery stent implantation was higher in the dissection group (81.2% vs. 8.9%, *p* < 0.001). No intergroup differences were observed in other variables (sex, diabetes, dyslipidemia, admission NIHSS score, anterior or posterior circulation involvement, intracranial/extracranial occlusion or intravenous thrombolysis, mechanical thrombectomy technique, number of passes, intracranial primary balloon angioplasty and stent placement, and extracranial primary balloon angioplasty; all *p* > 0.05) (Table 1).

**Table 1:** Clinical data and thrombus pathology: dissection vs. non-dissection groups.

Variable	Dissection (*n* = 16)	Non-dissection (*n* = 214)	*p*-value
Age, years, median (IQR)	45.0 (34.8–54.5)	66.0 (58.0–75.3)	<0.001
Male, *n* (%)	12 (75.0)	114 (53.3)	0.092
Hypertension, *n* (%)	2 (12.5)	114 (53.5)	0.002
Diabetes, *n* (%)	1 (6.2)	34 (15.9)	0.477
Dyslipidemia, *n* (%)	5 (31.2)	63 (29.4)	1.000
Previous stroke, *n* (%)	0 (0)	41 (19.2)	0.083
Atrial fibrillation, *n* (%)	0 (0)	108 (50.5)	<0.001
Smoking, *n* (%)	8 (50.0)	85 (39.7)	0.419
Alcohol, *n* (%)	5 (31.2)	51 (23.8)	0.548
Admission NIHSS score, median (IQR)	12.0 (8.5–22.5)	16.0 (12–21.3)	0.211
Anterior/posterior, *n* (%)			
Anterior	14 (87.5)	184 (86.0)	1.000
Posterior	2 (12.5)	27 (12.6)	
Both	0 (0)	3 (1.4)	
Intracranial/extracranial, *n* (%)			
Intracranial arteries	0	169 (79.0)	<0.001
Extracranial arteries	0	6 (2.8)	
Tandem lesion	16 (100)	39 (18.2)	
IVT, *n* (%)	6 (37.5)	69 (32.2)	0.665
Mechanical thrombectomy			
Stent retriever alone, *n* (%)	0	21 (9.8)	0.373
Contact aspiration alone, *n* (%)	6 (37.5)	75 (35.0)	0.843
Combined, *n* (%)	10 (62.5)	118 (55.1)	0.568
Number of passes, median (IQR)	2 (1–3)	2 (1–3)	0.394
Angioplasty (intracranial artery), *n* (%)			
Primary balloon angioplasty	0	26 (12.1)	0.227
Stent placement	0	16 (7.5)	0.610
Angioplasty (extracranial artery), *n* (%)			
Primary balloon angioplasty	3 (18.8)	14 (6.5)	0.103
Stent placement	13 (81.2)	19 (8.9)	<0.001
Reperfusion (eTICI) at end of procedure, *n* (%)			
2b50/2c/3	15 (93.8)	201 (93.9)	1.000
2c/3	8 (50.0)	159 (74.3)	0.045
Histopathology, median (IQR)			
RBC (%)	55.9 (39.7–65.6)	49.8 (32.7–63.1)	0.115
F/O (%)	38.6 (30.4–55.8)	45.9 (32.7–64.3)	0.121
WBC (%)	3.5 (2.7–5.4)	3.9 (2.7–5.0)	0.932
Total thrombus area (mm^2^)	31.3 (14.0–42.3)	15.8 (8.4–36.9)	0.103

IVT, intravenous thrombolysis; RBC, red blood cell; F/O, fibrin/other component; WBC, white blood cell; eTICI, expanded thrombolysis in cerebral infarction score; IQR, interquartile range.

Subgroup analyses identified distinct phenotypic and procedural signatures in stroke etiology. The patients with arterial dissection were the youngest (vs. LAA/CE/SUE; all *p* < 0.05), with dissection and LAA showing a higher male prevalence than did CE (*p* < 0.05). Vascular risk factors followed etiology-specific gradients: hypertension decreased stepwise (LAA > CE > dissection; *p* < 0.05), whereas atrial fibrillation was most frequent in the CE group (vs. LAA/SUE/dissection; all *p* < 0.001). The lifestyle factors differed as follows: smoking (LAA > CE, *p* < 0.05) and alcohol use (LAA > CE/SUE, *p* < 0.05) increased in the LAA group. The NIHSS score was lower in the LAA group than in the CE group (*p* < 0.05) but was comparable between the LAA and dissection/SUE groups. Procedural variations included higher stent retriever use in the LAA group (vs. dissection, CE, or SUE; *p* < 0.05) and increased intracranial balloon angioplasty/stenting in the LAA vs. non-LAA group (*p* < 0.05). Extracranial stenting peaked in dissection (vs. all groups; *p* < 0.01), whereas balloon angioplasty was dominant in LAA (vs. CE/SUE; *p* < 0.001) and dissection (vs. CE; *p* = 0.023). Supplementary Table 1 shows the clinical comparisons.

**Figure 3:**
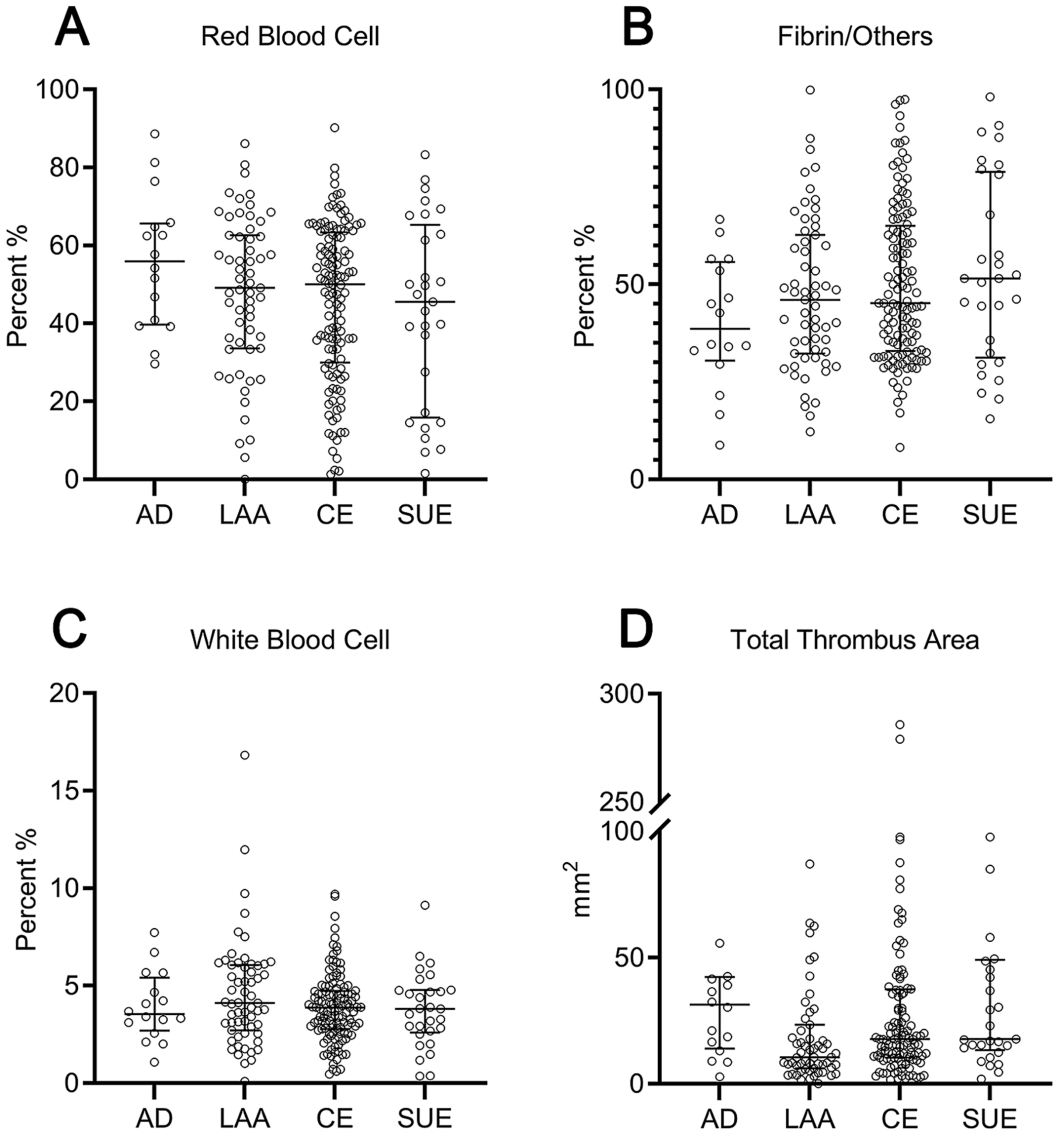
Scatter plot comparison of thrombus components and area across different etiologies. **(A)** Red blood cell proportion; **(B)** fibrin/others proportion; **(C)** white blood cell proportion; **(D)** total thrombus area. AD, arterial dissection; LAA, large artery atherosclerosis; CE, cardioembolic; SUE, stroke of undetermined etiology.

Histopathological analyses revealed no significant differences between the dissection and non-dissection groups in the RBC% (55.9% vs. 49.8%), F/O% (38.6% vs. 45.9%), WBC% (3.5% vs. 3.9%), or total thrombus area (31.3 mm^2^ vs. 15.8 mm^2^) (*p* > 0.05). Similarly, subgroup comparisons revealed no significant differences in the RBC%, F/O%, and WBC% (*p* > 0.05). The total thrombus area was significantly smaller in the LAA group (10.5 mm^2^) than in the CE (17.7 mm^2^) and dissection groups (31.3 mm^2^) (*p* < 0.05). There was no notable difference in the total thrombus area between the dissection and CE groups (*p* > 0.05) (Table 2 and Figure 3).

**Table 2:** Comparison of thrombus pathological characteristics among patients with different stroke etiologies.

Variable	Dissection (*n* = 16)	LAA (*n* = 59)	CE (*n* = 124)	SOE (*n* = 2)^a^	SUE (*n* = 29)	*p*-value
RBC (%), median (IQR)	55.9 (39.7–65.6)	49.1 (33.7–62.6)	50.1 (30.0–63.4)	67.3 (50.7–83.8)	45.5 (15.8–65.3)	0.344
F/O (%), median (IQR)	38.6 (30.4–55.8)	46.0 (32.2–62.7)	45.2 (32.9–65.1)	30.6 (15.6–45.6)	51.5 (31.2–78.9)	0.306
WBC (%), median (IQR)	3.5 (2.7–5.4)	4.1 (2.7–6.1)	3.9 (2.8–4.7)	2.2 (0.6–3.7)	3.8 (2.6–4.8)	0.502
Total thrombus area (mm^2^), median (IQR)	31.3 (14.0–42.3)	10.5 (6.1–23.4)	17.7 (10.4–37.4)	38.8 (2.2–75.4)	17.7 (13.3–49.1)	0.004

LAA, large artery atherosclerosis; CE, cardioembolism; SOE, stroke of other determined etiology (confirmed non-dissection causes); SUE, stroke of undetermined etiology; RBC, red blood cell; F/O, fibrin/other component; WBC, white blood cell.

a Groups with SOE (*n* = 2) were excluded from statistical comparisons owing to methodological limitations.

## 4 Discussion

Arterial dissection is a mechanical vascular injury caused by an intimal tear that creates a false lumen, leading to local thrombus formation and potential distal embolization. Dissection involves different mechanisms than atherothrombotic events resulting from plaque rupture or cardioembolic events resulting from atrial fibrillation. In the present study, distinct risk profiles were observed among the patient groups: patients with arterial dissection were the youngest and had the least hypertension; patients with LAA had strong associations with vascular risk factors such as hypertension, smoking, and alcohol use; and patients with CE showed the highest rates of atrial fibrillation. These patterns align with their respective pathophysiological mechanisms—mechanical or genetic in dissection, atherosclerosis in LAA, and cardiac embolism in CE—highlighting the need for targeted prevention and treatment strategies.

Previous studies on thrombus composition and stroke etiology have predominantly focused on CE, LAA, and SUE sources (4, 10). However, the findings have been inconsistent. Kim et al. reported that clots from CE have a significantly higher proportion of RBCs and lower proportion of fibrin than those from LAA (11). In contrast, another study reported that LAA thrombi had the highest percentages of RBCs, CE, and SUE (12). The discrepancies in these findings may be attributed to the limited statistical power in previous studies, which often resulted from small sample sizes. Furthermore, the nonuniform distribution of thrombus components may undermine the representativeness of individual histological sections, especially when sample sizes are insufficient. It has been suggested that, for compositional analyses in large-scale studies, one thrombus section per case is adequate, whereas three sections per case have been recommended in smaller cohorts (8). Variations in image analysis software may also contribute to discrepancies in results. Thrombi associated with arterial dissection remain underexplored. The Stroke Thromboembolism Registry of Imaging and Pathology study (13), which examined thrombi from 1,350 patients with AIS using Martius Scarlet Blue staining, reported a higher RBC content in arterial dissection thrombi than in those from LAA and CE (50% vs. 46% vs. 42%; *p* < 0.01). However, this study primarily assessed the compositional differentiation between LAA and CE thrombi rather than the diagnostic utility for arterial dissection. Theoretically, arterial dissection thrombi may exhibit distinct compositional profiles compared to thrombi of other etiologies; however, the composition of arterial dissection thrombi is influenced by various factors, such as the extent of the intimal tear, hemodynamic conditions, and age of the thrombus, which likely contribute to their intrinsic heterogeneity. We observed a higher proportion of RBCs in the dissection thrombi than in the LAA and CE (55% vs. 49% vs. 50%). However, this difference was not statistically significant, implying that the conventional histological evaluation of cellular composition has limited efficacy in distinguishing stroke etiologies. Notably, RBC-rich thrombi tend to have a softer consistency and are more vulnerable to fragmentation, leading to distal embolization (1). This may explain the higher incidence of tandem lesions in the dissection group than in the non-dissection group observed in this study.

An assessment of thrombus burden is critically important for guiding endovascular treatment strategy selection and predicting clinical outcomes. Current standard evaluation methods primarily rely on non-contrast computed tomography or computed tomography angiography (14, 15); however, these radiologically estimated burdens lack validation for physically retrieved thrombi. Area analyses of representative sections of the retrieved thrombi in this study revealed that thrombi associated with LAA exhibited the smallest cross-sectional area, which was significantly smaller than those associated with CE or dissection. This may reflect differences in the context of thrombosis. LAA occlusions typically represent acute thrombosis superimposed on underlying stenosis, forming rapidly via platelet aggregation in a high-shear environment, resulting in compact, adherent thrombi, where even small volumes can cause complete occlusion. CE thrombi, however, form slowly under low-shear conditions, driven by thrombin-mediated coagulation with greater growth potential. Dissection-related thrombi develop within the false lumen, which may extend longitudinally, facilitating larger thrombus volumes. These observed differences in thrombus area may inform thrombectomy device selection; smaller LAA thrombi may be more amenable to stent retrievers, while larger CE or dissection thrombi might be better suited for aspiration thrombectomy. Notably, among the 88 patients excluded because of non-retrieved thrombi, 76 (86.4%) were LAA cases, suggesting a lower thrombus burden in the LAA. Rather than thrombus absence, these small thrombi may fragment and migrate distally; when advancing microcatheters/microwires through occluded arteries, fresh thrombus fragments can be pushed into patent distal segments, where endogenous lysis occurs (16). In such scenarios, successful reperfusion was achieved either by direct primary balloon angioplasty and/or stenting, despite mechanical thrombectomy attempts without thrombus retrieval. Furthermore, thrombi classified as SUE demonstrated a significantly larger mean area than did the LAA thrombi, providing evidence that potentially supports a non-LAA origin in a subset of SUE cases.

This study has several limitations: (1) Only retrieved thrombus portions were analyzed, and unretrieved thrombus fragments may have affected the results; (2) the spatial heterogeneity (17) of thrombi may have limited the representativeness of two-dimensional sections for the overall composition; and (3) H&E staining cannot reliably differentiate fibrin from platelets, and it is unclear whether these components differ between arterial dissection and other causes, necessitating further immunohistochemical validation.

## 5 Conclusion

This study demonstrated the absence of significant compositional differences between the thrombi associated with arterial dissection-related stroke and those associated with non-dissection stroke. The findings suggest that conventional histopathological staining has limited efficacy in distinguishing dissection from other etiologies. Notably, the thrombi resulting from dissection, CE, and SUE exhibited significantly larger areas than those resulting from LAA, indicating a greater thrombus burden. These findings may have important implications for the optimization of mechanical thrombectomy strategies.

## Data Availability

The raw data supporting the conclusions of this article will be made available by the authors, without undue reservation.

## References

[1] Jolugbo P Ariëns RAS. Thrombus composition and efficacy of thrombolysis and thrombectomy in acute ischemic stroke. Stroke. (2021) 52:1131–42. doi: 10.1161/STROKEAHA.120.032810, PMID: 33563020 PMC7610448

[2] Kashihara K Nakai M Koga M Handa A Kobayashi S Usumoto S. Clinical characteristics, risk factors, and outcomes of arterial dissection-associated stroke: a 21-year cohort study from the Japan stroke data bank. J Atheroscler Thromb. (2025). doi: 10.5551/jat.65517, PMID: 40159224 PMC12416958

[3] Staessens S De Meyer SF. Thrombus heterogeneity in ischemic stroke. Platelets. (2021) 32:331–9. doi: 10.1080/09537104.2020.1748586, PMID: 32310709

[4] Patil S Darcourt J Messina P Bozsak F Cognard C Doyle K. Characterising acute ischaemic stroke thrombi: insights from histology, imaging and emerging impedance-based technologies. Stroke Vasc Neurol. (2022) 7:353–63. doi: 10.1136/svn-2021-001038, PMID: 35241632 PMC9453827

[5] Adams HP Bendixen BH Kappelle LJ Biller J Love BB Gordon DL. Classification of subtype of acute ischemic stroke. Definitions for use in a multicenter clinical trial. TOAST. Trial of org 10172 in acute stroke treatment. Stroke. (1993) 24:35–41. doi: 10.1161/01.str.24.1.35, PMID: 7678184

[6] Compter A Schilling S Vaineau CJ Goeggel-Simonetti B Metso TM Southerland A. Determinants and outcome of multiple and early recurrent cervical artery dissections. Neurology. (2018) 91:e769–80. doi: 10.1212/WNL.0000000000006037, PMID: 30068628

[7] Debette S Mazighi M Bijlenga P Pezzini A Koga M Bersano A. ESO guideline for the management of extracranial and intracranial artery dissection. Eur Stroke J. (2021) 6:XXXIX–LXXXVIII. doi: 10.1177/23969873211046475, PMID: 34746432 PMC8564160

[8] Staessens S Fitzgerald S Andersson T Clarençon F Denorme F Gounis MJ. Histological stroke clot analysis after thrombectomy: technical aspects and recommendations. Int J Stroke. (2020) 15:467–76. doi: 10.1177/1747493019884527, PMID: 31679478

[9] Fitzgerald S Wang S Dai D Murphree DH Pandit A Douglas A. Orbit image analysis machine learning software can be used for the histological quantification of acute ischemic stroke blood clots. PLoS One. (2019) 14:e0225841. doi: 10.1371/journal.pone.0225841, PMID: 31805096 PMC6894878

[10] Goebel J Gaida BJ Wanke I Kleinschnitz C Koehrmann M Forsting M. Is histologic thrombus composition in acute stroke linked to stroke etiology or to interventional parameters? AJNR Am J Neuroradiol. (2020) 41:650–7. doi: 10.3174/ajnr.A6467, PMID: 32193192 PMC7144659

[11] Kim SK Yoon W Kim TS Kim HS Heo TW Park MS. Histologic analysis of retrieved clots in acute ischemic stroke: correlation with stroke etiology and gradient-echo MRI. AJNR Am J Neuroradiol. (2015) 36:1756–62. doi: 10.3174/ajnr.A4402, PMID: 26159515 PMC7968760

[12] Niesten JM van der Schaaf IC van Dam L Vink A Vos JA Schonewille WJ. Histopathologic composition of cerebral thrombi of acute stroke patients is correlated with stroke subtype and thrombus attenuation. PLoS One. (2014) 9:e88882. doi: 10.1371/journal.pone.0088882, PMID: 24523944 PMC3921255

[13] Brinjikji W Nogueira RG Kvamme P Layton KF Delgado Almandoz JE Hanel RA. Association between clot composition and stroke origin in mechanical thrombectomy patients: analysis of the stroke thromboembolism registry of imaging and pathology. J Neurointerv Surg. (2021) 13:594–8. doi: 10.1136/neurintsurg-2020-017167, PMID: 33722963 PMC9069417

[14] Zhu K Bala F Zhang J Benali F Cimflova P Kim BJ. Automated segmentation of intracranial thrombus on NCCT and CTA in patients with acute ischemic stroke using a coarse-to-fine deep learning model. AJNR Am J Neuroradiol. (2023) 44:641–8. doi: 10.3174/ajnr.A7878, PMID: 37202113 PMC10249699

[15] Mojtahedi M Bruggeman AE van Voorst H Ponomareva E Kappelhof M van der Lugt A. Value of automatically derived full thrombus characteristics: an explorative study of their associations with outcomes in ischemic stroke patients. J Clin Med. (2024) 13:1388. doi: 10.3390/jcm13051388, PMID: 38592252 PMC10932251

[16] Yi TY Chen WH Wu YM Zhang MF Zhan AL Chen YH. Microcatheter “first-pass effect” predicts acute intracranial artery atherosclerotic disease-related occlusion. Neurosurgery. (2019) 84:1296–305. doi: 10.1093/neuros/nyy183, PMID: 29790969

[17] Liu Y Brinjikji W Abbasi M Dai D Arturo Larco JL Madhani SI. Quantification of clot spatial heterogeneity and its impact on thrombectomy. J Neurointerv Surg. (2022) 14:1248–52. doi: 10.1136/neurintsurg-2021-018183, PMID: 34911736 PMC11178127

