# Supplementary material for: Quantitative histopathologic profiling of arterial dissection-related thrombi in acute ischemic stroke: etiological comparisons
Source: Front Neurol. 2025 Aug 20;16:1640562. doi: 10.3389/fneur.2025.1640562 (PMC12404956; doi:10.3389/fneur.2025.1640562)
Supplement: Supplementary file 1 [file Table_1.docx]

Supplementary Material

# Supplementary Figures and Tables

## Supplementary Figures


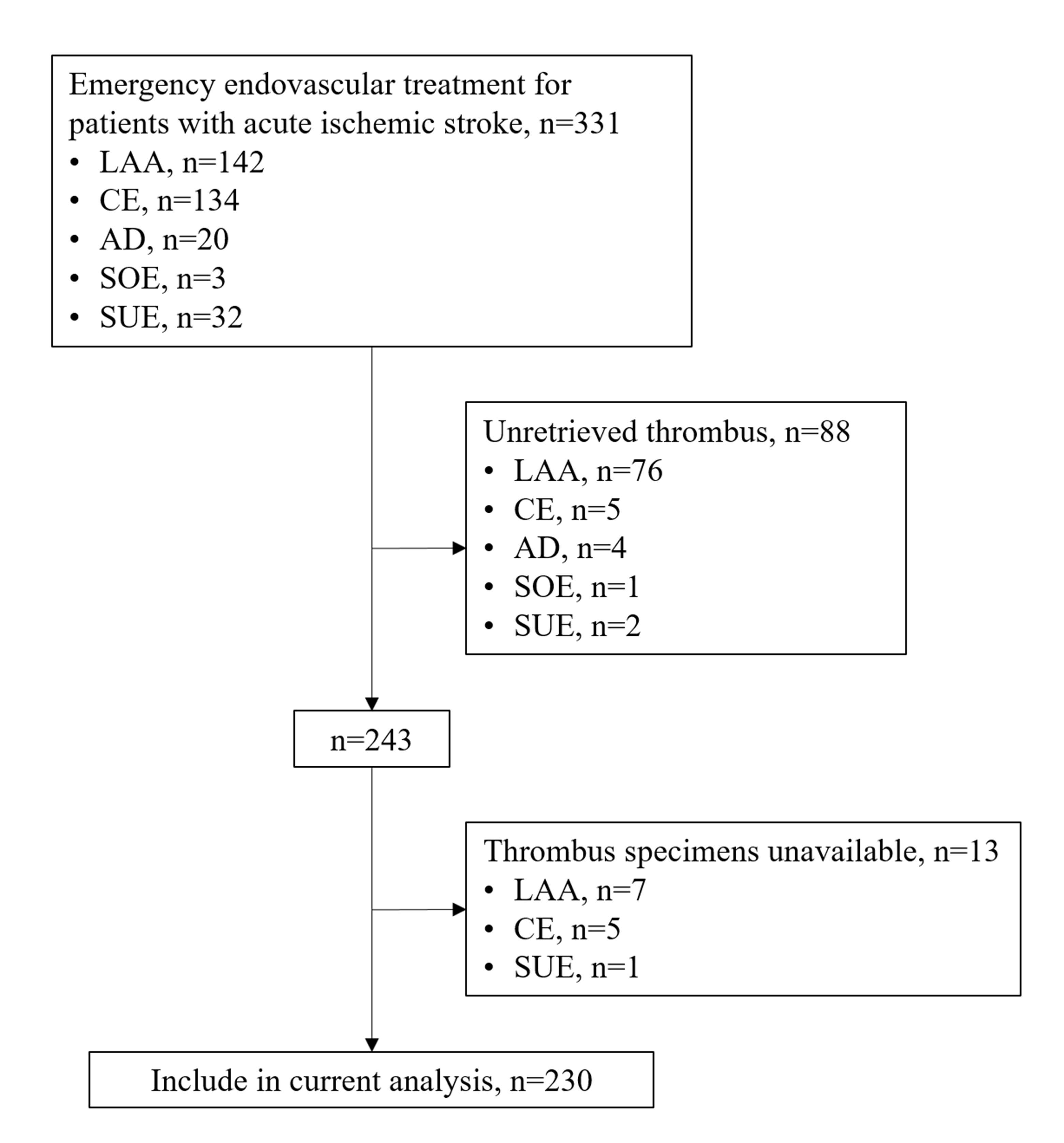


**Supplementary Figure 1.** **Flow diagram of patient selection.**

LAA, large-artery atherosclerosis; CE, cardioembolism; AD, arterial dissection; SOE, stroke of other determined etiology (confirmed non-dissection causes); SUE, stroke of undetermined etiology.

## Supplementary Tables 1.

| Variable | Dissection (n=16) | LAA(n=59) | CE(n=124) | SOE(n=2) ^a^ | SUE(n=29) | *p*-value |
| --- | --- | --- | --- | --- | --- | --- |
| Age, years, median (IQR) | 45.0(34.8-54.5) | 64.0(52.0-71.0) | 68.0(60.0-77.0) | 35.0(16.0-54.0) | 66.0(50.0-77.0) | ＜0.001 |
| Male, n (%) | 12 (75.0) | 47 (79.7) | 49 (39.5) | 1 (50.0) | 17 (58.6) | ＜0.001 |
| Hypertension, n (%) | 2 (12.5) | 43 (72.9) | 58 (46.8) | 0 (0) | 13 (44.8) | ＜0.001 |
| Diabetes, n (%) | 1 (6.2) | 10 (16.9) | 20 (16.1) | 1 (50.0) | 3 (10.3) | 0.741 |
| Dyslipidemia, n (%) | 5 (31.2) | 24 (40.7) | 33 (26.6) | 0 (0) | 6 (20.7) | 0.164 |
| Previous stroke, n (%) | 0 (0) | 18 (30.5) | 18 (14.5) | 1 (50.0) | 4 (13.8) | 0.010 |
| Atrial fibrillation, n (%) | 0 (0) | 2 (3.4) | 104 (83.9) | 0 (0) | 2 (6.9) | ＜0.001 |
| Smoking, n (%) | 8 (50.0) | 35 (59.3) | 37 (29.8) | 1 (50.0) | 12 (41.4) | 0.001 |
| Alcohol, n (%) | 5 (31.2) | 25 (42.4) | 20 (16.1) | 1 (50.0) | 5 (17. 2) | 0.001 |
| Admission NIHSS score, median (IQR) | 12.0(8.5-22.5) | 14.0(10.0-18.0) | 18.0(13.0-22.0) | 14.0(14.0-14.0) | 19.0(11.5-22.0) | 0.008 |
| **Anterior/Posterior, n (%)** |  |  |  |  |  |  |
| Anterior | 14 (87.5) | 45 (75.3) | 113 (91.1) | 2 (100.0) | 24 (82.8) | 0.025 |
| Posterior | 2 (12.5) | 14 (23.7) | 8 (6.5) | 0 (0) | 5 (17.2) |  |
| Both | 0 (0) | 0 (0) | 3 (2.4) | 0 (0) | 0 (0) |  |
| **Intracranial/Extracranial, n (%)** |  |  |  |  |  |  |
| Intracranial arteries | 0 (0) | 39 (66.1) | 106 (85.5) | 1 (50.0) | 23 (79.3) | ＜0.001 |
| Extracranial arteries | 0 (0) | 2 (3.4) | 4 (3.2) | 0 (0) | 0 (0) |  |
| Tandem lesion | 16 (100) | 18 (30.5) | 14 (11.3) | 1 (50.0) | 6 (20.7) |  |
| IVT, n (%) | 6 (37.5) | 18 (30.5) | 43 (34.7) | 0 (0) | 8 (27.6) | 0.833 |
| **Mechanical thrombectomy** |  |  |  |  |  |  |
| stent retriever alone, n (%) | 0 (0) | 16 (27.1) | 2 (1.6) | 1 (50.0) | 2 (6.9) | ＜0.001 |
| contact aspiration alone, n (%) | 6 (37.5) | 15 (25.4) | 50 (40.3) | 0 (0) | 10 (34.5) | 0.278 |
| Combined, n (%) | 10 (62.5) | 28 (47.5) | 72 (58.1) | 1 (50.0) | 17 (58.6) | 0.521 |
| Number of passes, median (IQR) | 2.0(1.0-3.0) | 2.0(1.0-2.0) | 2.0(1.0-3.0) | 2.5(1.0-4.0) | 2.0(1.0-3.5) | 0.110 |
| **Angioplasty (Intracranial artery), n** (%) |  |  |  |  |  |  |
| Primary balloon angioplasty | 0 (0) | 25 (42.4) | 1 (0.8) | 0 (0) | 0 (0) | ＜0.001 |
| Stent placement | 0 (0) | 15 (25.4) | 1 (0.8) | 0 (0) | 0 (0) | ＜0.001 |
| **Angioplasty (Extracranial artery), n** (%) |  |  |  |  |  |  |
| Primary balloon angioplasty | 3 (18.8) | 14 (23.7) | 0 (0) | 0 (0) | 0 (0) | ＜0.001 |
| Stent placement | 13 (81.2) | 17 (28.8) | 0 (0) | 0 (0) | 2 (6.9) | ＜0.001 |
| **Reperfusion (eTICI) at end of procedure, n (%)** |  |  |  |  |  |  |
| 2b50/2c/3 | 15 (93.8) | 56 (94.9) | 117 (94.4) | 2 (100.0) | 26 (89.7) | 0.690 |
| 2c/3 | 8 (50.0) | 44 (74.6) | 92 (74.2) | 2 (100.0) | 21 (72.4) | 0.231 |

**Supplementary Table 1. Comparison of baseline clinical data across different stroke etiologies.**

LAA, large-artery atherosclerosis; CE, cardioembolism; SOE, stroke of other determined etiology (confirmed non-dissection causes); SUE, stroke of undetermined etiology; IVT, intravenous thrombolysis; eTICI, expanded thrombolysis in cerebral infarction score; ^a^ Groups with SOE(n=2) were excluded from comparative statistical tests due to methodological constraints.
